# 일반병동 입원환자 급성 악화 예측을 위한 NEWS 확장 모델 예측력: 후향적 관찰 연구

**DOI:** 10.7586/jkbns.26.031

**Published:** 2026-08-26

**Authors:** Jae-kyeong Jo, Jinhwa Lee

**Affiliations:** 1 Ulsan University Hospital, Ulsan, Korea; 1 울산대학교병원; 2 Department of Nursing, University of Ulsan, Ulsan, Korea; 2 울산대학교 간호학과

**Keywords:** Clinical deterioration, Hospital rapid response team, Early warning score, Lactates, Bicarbonates, 급성 악화, 신속대응체계, 조기경고점수, 혈중 젖산, 중탄산염

## Abstract

**Background:**

This study evaluated whether an extended model combining the National Early Warning Score (NEWS) with blood lactate and serum total CO_2_ improves prediction of acute deterioration in adult general ward inpatients.

**Methods:**

The study analyzed electronic medical records from 437 patients admitted to a tertiary general hospital in Ulsan Metropolitan City from January 1 to December 31, 2024. Data were extracted from electronic medical records using a literature-informed data collection form. Analyses included descriptive statistics, the chi-square test, the t-test, logistic regression, and receiver operating characteristic analysis with area under the receiver operating characteristic curve (AUROC) estimation, performed using SPSS version 24.0.

**Results:**

In the extended model, each 1-point increase in NEWS and each 1 mmol/L increase in blood lactate were associated with higher odds of unplanned intensive care unit (ICU) admission (odds ratio [OR] = 1.23 and 1.22, respectively), whereas each 1 mmol/L increase in serum total CO_2_ was associated with lower odds (OR = 0.94). For 7-day mortality, NEWS and blood lactate were associated with higher odds of death (OR = 1.17 and 1.16, respectively), whereas serum total CO_2_ was associated with lower odds (OR = 0.91). The AUROC increased from .69 to .74 (*p* = .009) for unplanned ICU admission and from .68 to .77 (*p* = .003) for 7-day mortality.

**Conclusion:**

Adding blood lactate and serum total CO_2_ to NEWS improved discrimination for unplanned ICU admission and 7-day mortality. The extended model may support clinical decision-making and timely rapid response team intervention in general ward settings.

## 서론

### 1. 연구 배경

신속대응체계(Rapid Response System)는 일반병동 입원환자에게 예상치 못한 임상적 악화 상황이 발생했을 때 즉각적으로 의학적 조치를 시행하여 병원 내 심정지, 비계획적 중환자실 입실, 사망 등의 부정적 결과를 예방하는 환자 안전 시스템이다[1,2]. 신속대응팀(Rapid Response Team)은 신속대응체계 내에서 환자의 건강 상태가 중대한 악화 상황으로 진행되기 전에 조기 개입하여 상태 악화를 예방하거나 개선할 수 있는 기회를 제공하는 등의 중심 역할을 수행한다[3,4]. 신속대응팀의 긍정적 임상 역할에 대해서는 체계적 문헌고찰에서도 보고하고 있는데, 환자의 임상 악화를 조기에 식별하고 신속히 개입함으로써 비계획적인 중환자실 입실과 병원 내 사망률을 감소시켜 환자의 예후를 향상시키는 데 중요한 역할을 하는 것으로 나타났다[5-7]. 국내에서도 16개 상급종합병원 자료를 분석한 결과, 신속대응팀을 24시간 상시 운영하는 것이 병원 발생 패혈증 환자의 생존율 향상과 관련된 것으로 나타나, 신속대응팀의 긍정적 영향이 확인되었다[8].

그러나 모든 임상현장에서 신속대응체계가 활성화되는 것은 아니다. 선행연구에 따르면, 신속대응팀의 개입이 필요했던 환자 중 20% 내외에서 환자 상태 악화의 조기 발견이 지연된 것으로 보고하고 있다[9,10]. 이는 의료진이 환자 상태 악화를 적절히 인지하지 못했음을 시사한다. 환자 상태 악화 인지가 지연되는 첫 번째 이유는, 의료진이 환자에게 나타나는 악화 징후가 중증 상태로 진행될 가능성이 있는지, 혹은 단순한 일시적 생리적 변동인지를 판단하기 어려운 상황이다[11]. 두 번째 이유는 환자가 복합적인 만성 질환을 가지고 있는 경우 기존 질환으로 인해 발생하는 지속적인 증상인지 지금과는 다른 새로운 악화의 징후인지를 분별하기 어려울 수 있다[12]. 이는 신속대응팀 활성화하는데 기준을 명확히 적용하는데 어려움이 존재할 수 있다는 것이며, 그 결과 의료진은 객관적 선별 기준보다 자신의 임상 경험과 직관에 의존한 개입 필요성을 판단하는 경향이 있음을 보여준다[13]. 결과적으로 악화되고 있는 환자를 위한 조기 개입 기회를 놓치게 되어 환자 안전은 물론 불필요한 응급 상황 발생과 비계획적 중환자실 입실 증가로 이어질 수 있다[9]. 이러한 문제를 개선하고 신속대응팀의 효과성을 높이기 위해서는, 환자의 상태 악화 가능성을 정량적으로 평가하고 고위험 환자를 적시에 선별할 수 있는 정확한 예측 도구가 필요하다[14].

조기경고점수(National Early Warning Score, NEWS)는 일반병동 입원환자의 심각한 악화 상황을 조기에 인식할 수 있는 도구로 개발되었다[15]. 2012년에 영국에서 다양한 연구 결과들을 통합하여 NEWS를 개발하였으며 현재까지 가장 많이 활용되고 있다[16]. NEWS는 일반병동 입원환자의 심정지, 비계획적 중환자실 입실, 단기 사망과 같은 중대한 예후를 조기에 감지하는 데 유용한 도구로 검증되었다[14,17]. 그러나 활력징후 기반의 NEWS는 수술 후 합병증의 위험에 처해 있는 환자의 복잡한 병태생리를 충분히 반영하지 못한다는 한계가 있으며[18,19], 환자의 비전형적인 악화 징후를 인지할 수 있을 정도의 민감도와 특이성이 부족하다는 보고도 있다[14]. 이러한 한계를 보완하기 위해 최근 연구들은 NEWS에 대사적 지표를 추가한 예측 모델을 제시하고 있다. Durantez-Fernandez 등[20]은 응급실 내원 환자를 대상으로 한 연구에서 NEWS2와 혈중 젖산을 결합한 모델이 단독 NEWS2보다 더 우수한 민감도와 특이도를 보인다고 보고하였다. 또한, Dadeh 등[21]이 중증 패혈증 환자를 대상으로 한 무작위 대조군 연구에서, 젖산 수치 등 대사 지표를 포함한 모델이 환자 예후를 더 잘 예측한다고 보고하였다. 최근에는 혈중 총 이산화탄소가 환자의 대사 상태를 반영하는 중요한 지표로 주목받고 있다. 혈중 총 이산화탄소는 환자의 대사성 산-염기 상태와 관련이 깊으며[22,23], 일부 연구에서는 낮은 총 이산화탄소가 입원환자의 단기 사망률과 유의한 관련이 있음이 보고되었다[24].

그러나 이러한 연구들은 대부분 중환자실 또는 응급실 환경을 중심으로 수행되었으며, 일반병동 입원환자를 대상으로 NEWS와 대사적 지표를 이용하여 그 예측력을 연구한 보고는 미흡하다. 따라서 본 연구는 혈중 젖산과 혈중 총 이산화탄소를 NEWS에 포함한 확장 모델을 통해 일반병동 입원환자의 비계획적 중환자실 입실과 7일 이내 사망 예측에 있어 NEWS 단독 모델보다 향상된 예측력을 보이는지를 평가하고자 한다. 이를 통해 신속대응팀 활성화의 기준이 되는 새로운 조기경고점수의 개발을 위한 기초자료를 제공하고자 한다.

### 2. 연구의 목적

본 연구는 일반병동 입원환자를 대상으로 NEWS, 혈중 젖산 및 혈중 총 이산화탄소와 비계획적 중환자실 입실 및 7일 이내 사망 간의 관련성을 분석하고, 혈중 젖산과 혈중 총 이산화탄소를 포함한 NEWS 확장모형의 예측력을 평가하고자 하는 것이며, 구체적인 연구 목적은 다음과 같다.

1) 일반병동 입원환자의 일반적 특성, NEWS, 혈중 젖산, 혈중 총 이산화탄소에 따른 비계획적 중환자실 입실군과 비입실군의 차이, 7일 이내 사망군과 생존군의 차이를 비교한다.

2) 일반병동 입원환자의 NEWS, 혈중 젖산, 혈중 총 이산화탄소가 비계획적 중환자실 입실 및 7일 이내 사망 간의 관련성을 확인한다.

3) NEWS 확장 모델의 예측력을 평가하기 위해 NEWS 단독 모델과 확장 모델의 일반병동 입원환자 비계획적 중환자실 입실과 7일 이내 사망 예측력을 비교한다.

## 연구 방법

### 1. 연구 설계

본 연구는 일반병동 입원환자를 대상으로 NEWS, 혈중 젖산 및 혈중 총 이산화탄소와 비계획적 중환자실 입실 및 7일 이내 사망 간의 관련성을 분석하고, 혈중 젖산과 혈중 총 이산화탄소를 포함한 NEWS 확장모형의 예측력을 평가하는 후향적 관찰연구이다. 이에 관찰연구 보고지침인 Strengthening the Reporting of Observational Studies in Epidemiology (STROBE)에 따라 보고하였다.

### 2. 연구 대상

본 연구 대상자는 울산광역시에 소재한 상급종합병원 일반병동에 입원한 환자 중 2024년 1월 1일부터 2024년 12월 31일까지 신속대응팀이 활성화된 만 18세 이상의 성인 환자로 전체 표본 수는 934명이었다. 신속대응팀의 활성화는 환자의 상태가 악화가 우려되어 병동 의료진이 수동적으로 신속대응팀을 호출한 경우와 간호사가 전자의무기록에 입력한 활력징후를 바탕으로 자동 산출된 NEWS에 근거하여 신속대응팀이 능동적으로 개입한 경우로 구분되며, 본 연구는 이 두 유형 모두를 연구에 포함하였다.

이 중 일반병동이 아닌 곳에 입원 중인 환자(16명), 24시간 이내에 혈중 젖산 및 혈중 총 이산화탄소 수치가 측정되지 않은 환자(289명), 심정지 발생 직후 병원 내 심폐소생술 방송에 의해 신속대응팀이 호출된 환자(28명), 연명의료중단 및 심폐소생술 거절 요청에 등록된 환자(128명)은 분석에서 제외하였다. 또한, 신속대응팀 활성화 기록지가 누락되어 주요 임상 정보에 대한 결측치가 있는 36명도 제외하여 총 437명이 최종 분석대상자였다(Appendix 1).

### 3. 연구 도구

#### 1) 일반적 특성

대상자의 일반적 특성은 성별, 만 나이, 주 진료과, 기저질환, 활성화 평가, 활성화 당시 활력징후, 활성화 당시 산소 적용 여부, 활성화 당시 의식 상태를 포함한다. 주 진료과는 내과, 외과로 크게 구분하였으며, 내과는 호흡기내과, 심장내과, 소화기내과, 감염내과, 혈액종양내과, 내분비내과, 류마티스내과, 신경과, 정신과, 가정의학과, 재활의학과, 신장내과와 부인과가 포함되었다. 외과는 일반외과, 흉부외과, 신경외과, 정형외과, 비뇨기과, 이비인후과, 치과가 포함되었다. 기저질환은 신속대응팀 간호사가 활성화 기록지에 기재한 정보를 바탕으로 수집하였으며, 해당 항목은 암, 당뇨병, 만성 폐질환, 심혈관 질환, 간담췌질환, 위장관 질환, 뇌혈관 질환, 만성 신장 질환, 갑상선 질환을 포함하였다. 활성화 평가는 신속대응팀 간호사가 활성화 이후 환자의 상태를 평가하여 기록한 항목으로 패혈증/패혈성 쇼크, 호흡 곤란, 쇼크, 부정맥, 갑작스러운 의식 변화, 대사성 산증, 저혈압이 해당된다. 활성화 당시의 활력징후, 산소 적용 여부, 의식 상태는 활성화 기록지를 기반으로 수집하였다. 수집 기준은 신속대응팀이 NEWS 기반으로 능동적으로 개입한 경우에는 병동 간호사가 전자의무기록에 입력한 자료를 기준으로 하였으며, 병동 의료진이 수동적으로 신속대응팀을 호출한 경우에는 신속대응팀 간호사가 현장에서 재측정 및 직접 관찰한 자료를 기준으로 하였다.

#### 2) 조기경고점수(NEWS)

NEWS는 최초 Morgan 등[25]이 1997년 활력징후를 기반으로 환자의 급성 악화를 조기에 인지하기 위한 제안한 Early Warning Score (EWS)를 기반으로 개발되었다. 이후, Subbe 등이 기존 EWS를 내과 입원환자를 대상으로 검증하며 Modified Early Warning Score (MEWS)로 수정하였으며[26], 2012년 영국 왕립내과학회(Royal College of Physicians, RCP)[27]가 병원 간 표준화된 조기경고체계로서 개발하였다.

NEWS는 간호사가 전자의무기록(Electronic Medical Record)에 입력된 호흡수, 산소포화도, 체온, 수축기 혈압, 맥박수, 의식 수준의 6가지 생리학적 변수와 산소 적용 여부를 평가 항목으로 점수를 산출한다. 호흡수는 흉곽의 움직임을 관찰하여 직접 측정하고, 산소포화도는 휴대용 산소포화도 측정기를 이용하여 측정한다. 체온은 고막체온계를 이용하고, 혈압은 자동 또는 수동 혈압계를 이용한다. 맥박 수는 자동혈압계 또는 심전도 감시 장치를 이용하여 확인한다. 산소포화도, 체온, 혈압 및 맥박수는 각 병동에서 정기적으로 교정 및 관리되는 표준 임상 측정 장비를 이용하여 측정하였다. 후향적 의무기록 연구의 특성상 개별 측정장비 모델명은 확인할 수 없었다.

NEWS는 Appendix 2와 같이 각 생리학적 변수는 이상 정도에 따라 0~3점이 부여되며, 산소 투여 시 추가 2점을 부여하여 총점을 계산한다. NEWS 총점은 최소 0점에서 20점 범위이며, 점수가 높을수록 환자의 급성 악화 위험이 증가한다. RCP는1~4점은 저위험군, 5~6점 또는 단일 항목 3점은 중등도 위험군, 7점 이상은 고위험군으로 분류하여 활용할 것을 권고하고 있다.

#### 3) 혈중 젖산

신속대응팀 활성화 시간을 기준으로 24시간 이전에 시행된 혈액 검사 중 혈중 젖산 수치(mmol/L)를 활용하였다. 동일 기간 내 반복 측정된 경우에는 활성화 시점과 시간 간격이 가장 짧은 값을 대푯값으로 선택하였다. 혈중 젖산 검사는 환자에게서 채취된 동맥혈을 이용하여 원내 진단검사실에 있는 기기(The epoc Blood Analysis System, Siemens Healthineers, Tarrytown, USA)를 이용하거나 신속대응팀이 소지하고 있는 현장검사기(Point-of-Care Testing)를 이용하여 검사한 결과를 전자의무기록에서 수집하였다.

#### 4) 혈중 총 이산화탄소

신속대응팀 활성화 시간을 기준으로 24시간 이전에 시행된 혈액 검사 중 혈중 총 이산화탄소 수치(mmol/L)를 활용하였다. 혈중 총 이산화탄소는 병동간호사가 환자 정맥혈에서 채취한 검체를 통해 통상적인 임상화학검사로 측정된 결과를 전자의무기록에서 수집하였다. 후향적 의무기록 연구의 특성상 개별 장비 모델명은 확인할 수 없었다.

#### 5) 비계획적 중환자실 입실

비계획적 중환자실 입실은 일반병동에 입원 중인 환자가 급성 악화 상태로 인해 사전에 계획되지 않은 상태에서 중환자실로 전동되는 것을 의미하며, 환자의 임상적 악화와 예후를 평가하는 중요한 지표로 보고하고 있다[28]. 이에 본 연구에서는 일반병동 입원환자의 신속대응팀 활성화 시간 이후를 기준으로 12시간 이내에 발생한 비계획적 중환자실 입실 여부를 분석에 포함하였다. 비계획적 중환자실 입실 여부는 중환자실 간호일지 내 ‘중환자실 입실함’으로 기록된 시간을 기준으로 정의하였으며, 중환자실에 입실한 경우는 1, 입실하지 않은 경우는 0으로 구분되는 이분형 구조로 코딩하였다.

#### 6) 7일 이내 사망

7일 이내 사망은 신속대응팀 활성화 이후 단기간 내 발생한 병원 내 사망을 의미하며, 환자의 단기 예후를 평가하는 대표적인 임상 결과 지표이다[29]. 본 연구에서는 신속대응팀 활성화 시간 이후를 기준으로 7일 이내에 발생한 사망 여부를 분석에 포함하였다. 7일 이내 사망 여부는 간호일지 내 ‘사망함’으로 기록된 시간을 기준으로 정의하였으며, 사망한 경우는 1, 생존한 경우는 0으로 코딩하였다.

### 4. 자료 수집

연구 대상자의 일반적 특성, NEWS, 혈중 젖산, 혈중 총 이산화탄소, 비계획적 중환자실 입실, 7일 이내 사망 관련 정보는 울산대학교병원의 전자의무기록과 신속대응팀 활성화 기록지를 기반으로 수집하였다. 연구 자료는 엑셀 파일 형태로 제공받았으며, 연구 참여자를 식별할 수 있는 개인정보는 모두 제거된 상태였다. 이후 연구 고유번호를 부여하여 비식별화 과정을 거쳤다. 입력된 데이터는 암호화된 엑셀 파일 형태로 저장하였고, USB 드라이브에 보관하였다. 저장된 자료에 대한 접근은 연구 책임자만 제한적으로 허용되었으며, 외부 네트워크와 차단된 저장 환경인 USB에 백업본을 별도로 보관함으로써 자료의 보안성을 강화하였다.

### 5. 자료 분석

본 연구는 SPSS 버전 24.0 (IBM Corp., Armonk, NY, USA)을 사용하여 다음과 같이 통계 분석하였다.

첫째, 대상자의 일반적 특성, NEWS, 혈중 젖산 및 혈중 총 이산화탄소에 따른 비계획적 중환자실 입실군과 비입실군 차이, 7일 이내 사망군과 생존군 차이 분석을 위해 범주형 변수는 모두 Pearson 카이제곱 검정으로 분석하였다. 기대빈도가 작은 경우 교차표에 따라 Fisher의 정확 검정 또는 Fisher-Freeman-Halton 정확 검정을 이용하여 분석하였다. 일반적 특성 중 기저질환은 복수응답 자료로 각 질환의 유무를 이분형 변수로 변환하여 분석하였다. 9개 기저질환에 대한 반복검정으로 인한 제1종 오류를 통제하기 위해 Bonferroni 보정을 적용하였으며, 보정된 유의수준은 *p* < .006이었다. 연속형 변수는 Shapiro-Wilk test로 정규성 검정을 먼저 시행하였다. 일반적 특성 중 연령은 정규성이 확보되어 독립표본 t-검정을 실시하였고, NEWS, 혈중 젖산 및 혈중 총 이산화탄소는 정규성이 위배되어 Mann-Whitney U 검정을 이용하여 분석하였다.

둘째, 대상자의 NEWS, 혈중 젖산, 혈중 총 이산화탄소 수치와 비계획적 중환자실 입실 여부 및 7일 이내 사망 간의 관련성을 분석하기 위해 로지스틱 회귀분석을 시행했다. 비계획적 중환자실 입실 및 7일 이내 사망 간의 관련성을 분석하기 위한 Model 1은 NEWS만을, Model 2는 NEWS, 혈중 젖산, 혈중 총 이산화탄소를 독립변수로 포함하였다. 모델의 적합도는 Hosmer-Lemeshow goodness-of-fit test로 평가하였으며, 모델의 설명력은 Cox & Snell R^2^와 Nagelkerke R^2^를 사용하였다. 로지스틱 회귀모형의 과적합 가능성을 검토하기 위해 사건 수 대비 변수 수(events per variable, EPV)를 산출하였으며, EPV 10 이상을 참고 기준으로 하였다.

셋째, 대상자의 NEWS, 혈중 젖산, 혈중 총 이산화탄소를 기반으로 비계획적 중환자실 입실과 7일 이내 사망에 대한 예측력을 ROC 곡선하면적(area under the receiver operating characteristic curve, AUROC)을 통해 평가하였다. 비계획적 중환자실 입실과 7일 이내 사망을 예측하기 위해 Model 1 (NEWS 단독)과 Model 2 (NEWS 확장 모델: NEWS, 혈중 젖산, 혈중 총 이산화탄소 포함)로 로지스틱 회귀모형을 구축하였다. 각 모형에서 산출된 회귀계수와 상수를 이용한 로지스틱 회귀식으로부터 개별 대상자의 예측확률을 산출하였으며, 산출된 예측확률로부터 ROC 곡선과 AUROC를 산출하였다. AUROC 차이는 SPSS 대응표본 ROC 분석을 이용하였고, 통계적 유의성은 Z 통계량을 이용하여 검정하였다. 그 외 절단점, 민감도, 특이도를 통해서도 비교하였다.

### 6. 윤리적 고려

본 연구의 자료수집은 울산대학교병원 생명윤리심의위원회(Institutional Review Board)의 심의 및 승인(File No: 2025-01-031)을 받은 후 시행하였다. 제공받은 연구 자료는 엑셀파일 형태였고, 개인 정보가 제거된 비식별화된 데이터로서 대상자의 익명성과 기밀성을 유지하였다. 수집된 자료는 지정된 연구자 외에는 접근할 수 없도록 별도의 드라이브에 잠금장치로 보안을 유지하고 비밀번호는 연구자가 직접 관리하였다. 수집된 자료는 연구 종료 3년 후 즉시 폐기할 것이다.

## 연구 결과

### 1. 일반적 특성, NEWS, 혈중 젖산, 혈중 총 이산화탄소에 따른 비계획적 중환자실 입실 차이

연구 대상자의 일반적 특성, NEWS, 혈중 젖산, 혈중 총 이산화탄소에 따른 비계획적 중환자실 입실군과 비입실군 차이를 비교한 결과는 Table 1과 같다. 신속대응팀 활성화 평가에서 비계획적 중환자실 입실군과 비입실군 간에 통계적으로 유의한 차이를 보였으며(χ^2^ = 40.90, *p* < .001), 특히 입실군에서 패혈증/패혈성 쇼크(58.1%), 대사성 산증(57.4%)의 비율이 높게 나타났다.

NEWS의 중앙값은 입실군에서 9.00 (6.00-11.00)점, 비입실군에서 6.00 (5.00-9.00)점으로 입실군이 통계적으로 유의하게 더 높았다(U = 13230.50, *p* < .001). 혈중 젖산은 입실군 2.17 (1.06-4.12) mmol/L, 비입실군 1.44 (0.90-2.09) mmol/L으로 입실군의 혈중 젖산이 통계적으로 유의하게 더 높았다(U = 15665.00, *p* < .001). 반면, 혈중 총 이산화탄소는 입실군 20.00 (17.00-23.50) mmol/L, 비입실군 22.00 (19.00-25.00) mmol/L으로 입실군의 혈중 총 이산화탄소가 비입실군에 비해 통계적으로 유의하게 낮았다(U = 16108.00, *p* < .001).

### 2. 일반적 특성, NEWS, 혈중 젖산, 혈중 총 이산화탄소에 따른 7일 이내 사망 여부 차이

연구 대상자의 일반적 특성, NEWS, 혈중 젖산, 혈중 총 이산화탄소에 따른 7일 이내 사망군과 생존군의 차이를 비교한 결과는 Table 2와 같다. 주 진료과에서 7일 이내 사망군과 생존군 간에 통계적으로 유의한 차이를 보였으며(χ^2^ = 6.37, *p* = .012), 7일 이내 사망군의 모든 대상자가 내과 환자(100%)로 외과 환자는 없었다.

NEWS의 중앙값은 사망군 9.00 (7.00-11.00)점, 생존군 7.00 (5.00-9.00)점으로 사망군의 점수가 통계적으로 유의하게 더 높았다(U = 5,212.00, *p* < .001). 혈중 젖산은 사망군 2.60 (1.48-5.38) mmol/L, 생존군 1.46 (0.90-2.36) mmol/L으로 사망군의 혈중 젖산이 통계적으로 유의하게 더 높았다(U = 4,690.50, *p* < .001). 반면, 혈중 총 이산화탄소는 사망군 18.00 (15.00-22.00) mmol/L, 생존군 22.00 (19.00-25.00) mmol/L으로 사망군의 혈중 총 이산화탄소가 생존군에 비해 통계적으로 유의하게 낮았다(U = 5,129.50, *p* < .001).

### 3. 일반병동 입원환자의 비계획적 중환자실 입실 및 7일 이내 사망 간의 관련성

일반병동 입원환자의 NEWS, 혈중 젖산, 혈중 총 이산화탄소와 비계획적 중환자실 입실 및 7일 이내 사망 간의 관련성을 확인하기 위해 로지스틱 회귀분석을 실시한 결과는 Table 3과 같다. 비계획적 중환자실 입실 및 7일 이내 사망과의 관련성을 분석하기 위한 Model 1은 NEWS를, Model 2는 NEWS, 혈중 젖산, 혈중 총 이산화탄소를 독립변수로 포함하였다.

비계획적 중환자실 입실 및 7일 이내 사망 Model 2의 -2 log likelihood 값이 각각 483.40, 236.18으로 Model 1의 값 510.50, 257.47보다 감소하여 모형의 적합도가 향상되었다. Hosmer–Lemeshow 적합도 검정 결과, 비계획적 중환자실 입실의 Model 1과 Model 2는 각각 *p* = .891과 *p* = .614였으며, 7일 이내 사망의 Model 1과 Model 2는 각각 *p* = .523과 *p* = .129으로 나타나 모든 모형에서 관찰값과 예측값 간 유의한 차이가 없어 적합도가 수용 가능한 것으로 나타났다. Cox & Snell R²과 Nagelkerke R² 분석 결과, 비계획적 중환자실 입실 Model 1은 각각 .10, .14에서 Model 2 .15, .21로 증가하였으며, 7일 이내 사망의 경우에도 Model 1에서는 .03, .08에서 .07, .17로 증가하였다. EPV는 비계획적 중환자실 입실의 Model 1과 Model 2에서 각각 77.00과 25.67이었고, 7일 이내 사망의 Model 1과 Model 2에서는 각각 41.00과 13.67로 모든 모형에서 참고 기준인 10 이상이었다.

일반병동 입원환자의 비계획적 중환자실 입실 Model 1에서는 NEWS가 1점 증가할 때마다 비계획적 중환자실 입실 오즈는 1.25배 높았다(95% CI: 1.16-1.34). Model 2에서는 NEWS가 1점 증가할 때마다 비계획적 중환자실 입실 오즈는 1.23배 높았고(95% CI: 1.14-1.32), 혈중 젖산 수치는 1 mmol/L 증가할 때마다 비계획적 중환자실 입실 오즈는 1.22배 높았다(95% CI: 1.08-1.37). 반면, 혈중 총 이산화탄소 수치는 1 mmol/L 증가할 때마다 비계획적 중환자실 입실 오즈는 6% 낮았다(95% CI: 0.90-0.99).

7일 이내 사망 Model 1에서는 NEWS가 1점 증가할 때마다 7일 이내 사망 오즈는 1.20배 높았다(95% CI: 1.09-1.32). Model 2에서는 NEWS가 1점 증가할 때마다 7일 이내 사망 오즈는 1.17배 높았고(95% CI: 1.06-1.29), 혈중 젖산 수치는 1 mmol/L 증가할 때마다 7일 이내 사망 오즈는 1.16배 높았다(95% CI: 1.03-1.31). 반면, 혈중 총 이산화탄소 수치는 1 mmol/L 증가할 때마다 7일 이내 사망 오즈는 9% 낮았다(95% CI: 0.85-0.98). 

### 4. 비계획적 중환자실 입실과 7일 이내 사망을 예측하기 위한 NEWS 확장 모델 예측력 분석

**Figure 1.**
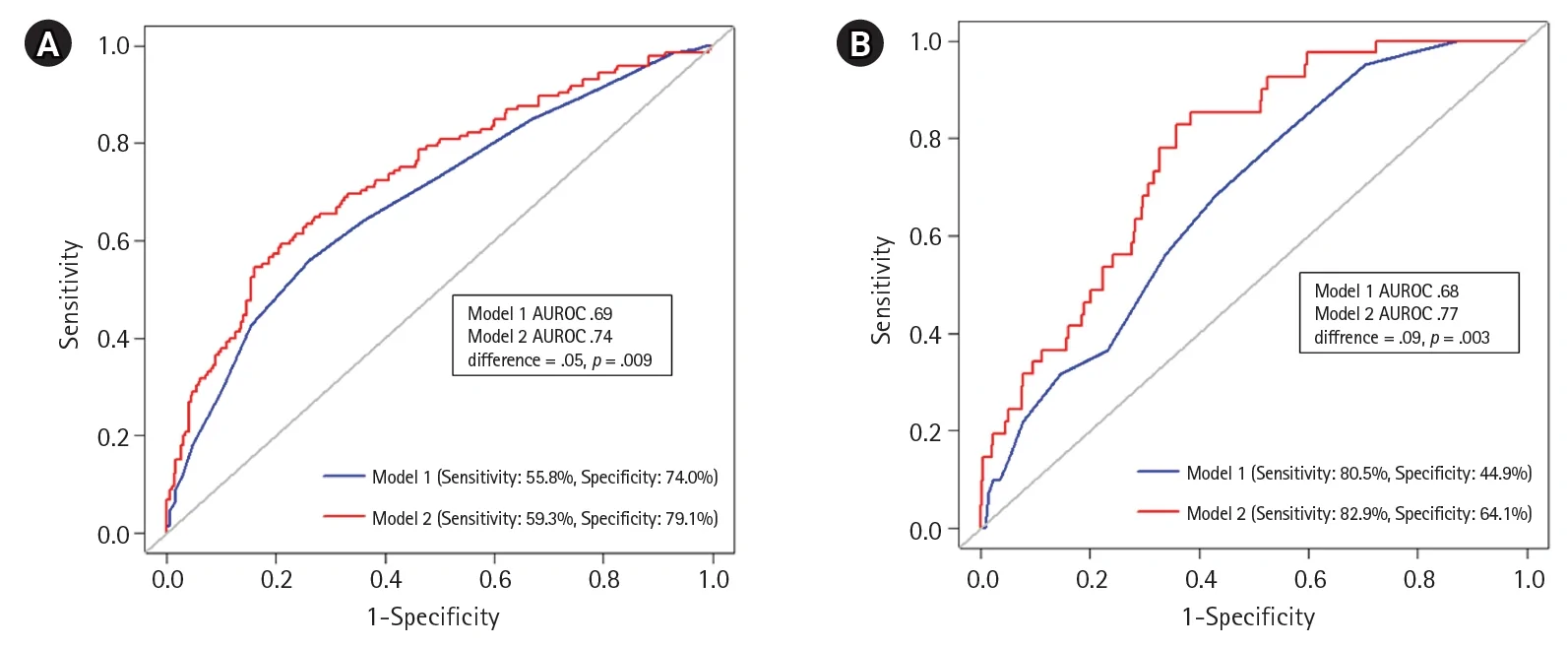
Predictive performance of the extended NEWS model for unplanned intensive care unit admission and 7-day mortality. (A) Analysis of the sensitivity and specificity of the extended NEWS model for predicting unplanned intensive care unit admission. (B) Analysis of the sensitivity and specificity of the extended NEWS model for predicting 7-day mortality. AUROC = Area under the receiver operating characteristic curve; NEWS = National Early Warning Score.

비계획적 중환자실 입실과 7일 이내 사망에 대한 예측 지표로서 Model 1 (NEWS 단독)과 Model 2 (NEWS 확장 모델: 혈중 젖산, 혈중 총 이산화탄소 포함)의 예측력을 AUROC로 비교 분석한 결과는 Table 4, Figure 1과 같다. 각 모델의 로지스틱 회귀식으로부터 개별 대상자의 예측확률을 산출하였으며, 산출된 예측확률을 이용하여 ROC 곡선과 AUROC를 산출하였다. 그 결과, NEWS 확장 모델인 Model 2가 NEWS를 단독으로 분석한 Model 1에 비해 비계획적 중환자실 입실과 7일 이내 사망에 대해 더 높은 예측력을 보였다.

비계획적 중환자실 입실 예측에서 Model 1의 최적 NEWS 절단점은 8.50이었고, 민감도는 55.8%, 특이도는 74.0%였다. Model 2의 최적 예측확률 절단점은 0.37이었으며, 민감도와 특이도는 각각 59.3%와 79.1%였다. Model 2의 AUROC는 .74 (95% CI: 0.69–0.79)로 Model 1의 .69 (95% CI: 0.63–0.74)보다 유의하게 높았다(ΔAUROC = .05, *p* = .009).

7일 이내 사망 예측에서 Model 1의 최적 NEWS 절단점은 6.50이었고, 민감도는 80.5%, 특이도는 44.9%였다. Model 2의 최적 예측확률 절단점은 0.08이었으며, 민감도와 특이도는 각각 82.9%와 64.1%였다. Model 2의 AUROC는 .77 (95% CI: 0.71–0.83)로 Model 1의 .68 (95% CI: 0.60–0.76)보다 유의하게 높았다(ΔAUROC = .09, *p* = .003).

## 논의

본 연구는 일반병동 입원환자를 대상으로 NEWS, 혈중 젖산, 혈중 총 이산화탄소가 비계획적 중환자실 입실 및 7일 이내 사망 간의 관련성을 파악하고자 시도되었다. 더불어 혈중 젖산과 혈중 총 이산화탄소를 NEWS에 포함한 확장 모델을 통해 비계획적 중환자실 입실과 7일 이내 사망 예측에 있어 NEWS 단독 모델보다 향상된 예측력을 보이는지를 평가하여, 새로운 조기경고점수 개발을 위한 근거 자료를 제공하고자 하였다.

본 연구 결과, NEWS와 혈중 젖산, 혈중 총 이산화탄소 수치는 비계획적 중환자실 입실 및 7일 이내 사망 가능성에 통계적으로 유의하게 관련성이 있었다. NEWS는 기존 연구들에서 입원환자의 예후 예측 및 중환자실 입실 위험, 사망률과 밀접한 관련이 있음이 반복적으로 보고된 바 있다[14,16]. NEWS가 7점 이상일 경우 환자의 사망률과 비계획적 중환자실 입실이 통계적으로 유의하게 증가한다고 보고하였고[15], NEWS를 반복 측정하여 점수변화의 추이를 살펴보면, 병원 내 사망을 더 잘 설명할 수 있다고 보고하였다[17]. 특히, 일반병동 입원환자의 급성 악화 대부분이 호흡기 이상에서 시작되는데, NEWS는 호흡수, 산소포화도, 산소 적용 여부를 기반으로 점수가 산출되므로 호흡기 악화를 더 민감하게 반영할 수 있다[29].

혈중 젖산은 임상 현장에서 저산소증의 조기 지표로 널리 활용되고 있으며, 고젖산혈증은 중증도 및 사망 위험 증가와 밀접한 연관이 있음을 다양한 선행연구에서 보고하고 있다. 혈중 젖산이 4.0 mmol/L 이상일 경우, 이는 중증 패혈증이나 패혈성 쇼크를 나타내며, 병원 내 사망률 증가와 유의한 연관성이 있다고 보고하였고[30,31], 중증 감염 환자를 대상으로 한 7편의 무작위 대조 연구를 분석한 결과에서도 혈중 젖산이 낮은 군에 비해 높은 군이 병원 내 사망률이 유의하게 높은 것으로 나타나 본 연구 결과와 맥락을 같이 한다[32]. 혈중 젖산이 높은 상태가 지속되면, 인체 조직에 산소가 부족한 저산소증이 이어지게 되고, 이로 인한 생리적 불균형이 치료 반응 부진과 사망률 증가로 이어질 수 있음을 시사하였다[33].

본 연구 결과, 혈중 총 이산화탄소는 1 mmol/L 증가할 때마다 환자의 비계획적 중환자실 입실과 7일 이내 사망 가능성이 감소하는 것으로 확인되었다. 이는 혈중 총 이산화탄소가 낮은 경우에는 대사성 산증과 같은 산-염기 불균형의 간접적인 지표가 될 수 있음을 시사한다[22]. Kim 등[24]의 연구에서 혈중 총 이산화탄소가 22 mmol/L 이하인 환자에서 주요 장기 기능 저하, 신대체요법 필요성 증가 등이 관찰되었고, 수치가 더 낮아질수록 사망 위험이 1.7배 증가했다. 또한 Han 등[34]의 연구에서도 혈중 총 이산화탄소 수치가 23 mmol/L 미만일 경우 28일 사망 위험이 통계적으로 유의하게 높아진다고 보고하고 있어 본 연구 결과와 일치한다.

본 연구 결과, NEWS와 혈중 젖산, 혈중 총 이산화탄소를 포함한 확장모델이 일반병동 입원환자의 비계획적 중환자실 입실 및 7일 이내 사망 예측에 있어 NEWS 단독모델보다 우수한 예측 성능을 보이는 것으로 나타났다. NEWS는 환자의 급성 악화를 조기 발견하고, 의료진 간에 급성 악화를 표준화하기 위한 도구로 개발되었지만, 최근에는 다양한 기저질환과 관련된 서로 다른 급성 악화 상황을 완전히 설명하기에는 한계가 있는 것으로 보고되고 있다[15]. 실제 영국 환자를 대상으로 한 연구에서 NEWS의 AUROC가 내과 환자의 72시간 사망과 비계획적 중환자실 입실을 예측하는데 좋은 성능을 보이는 것으로 보고하였지만, 이것만으로 최종 의사결정을 하는 것은 한계가 있다고 보고하였다[35]. 또한 대사 이상이 이미 진행되어도 NEWS 점수가 낮게 나올 수 있기 때문에 다른 바이오마커들을 활용하는 것이 필요하다고 하였다[14,35]. 아직 NEWS와 혈중 젖산, 혈중 총 이산화탄소를 모두 포함한 모델을 활용한 연구를 찾을 수는 없었지만, 대사 스트레스를 의미하는 혈중 젖산을 포함한 NEWS 모델의 예측율을 분석하는 연구들은 상당히 진행되고 있었다. Durantez-Fernandez 등[20]은 응급실 환자를 대상으로 NEWS 단독, 혈중 젖산 단독, NEWS2와 혈중 젖산을 결합한 모델의 2일 사망 예측을 비교한 결과 NEWS2-L 모델이 AUROC 0.923으로 가장 높은 예측력을 보였다고 보고하였다. Dadeh 등[21]은 패혈증 의심 환자의 NEWS와 혈중 젖산을 포함한 NEWS-L의 2일 사망, 중환자실 치료 필요도를 ROC로 비교한 결과 NEWS-L이 더 잘 예측하는 것으로 보고하였다. 최근 중국에서 응급실 내원 환자 중 비계획적으로 중환자실에 입실한 환자를 대상으로 한 연구가 시행되었는데, MEWS와 혈중 총 이산화탄소를 결합한 모델이 MEWS를 단독 사용하는 것에 비해 병원 내 사망을 더 잘 예측하는 것으로 보고하고 있어 본 연구 결과가 맥락을 같이 하고 있다[36]. NEWS는 주로 임상적으로 관찰 가능한 생리적 변화를 반영하는 반면, 혈중 젖산은 조직 저관류, 세포 대사 이상과 관련된 변화를, 혈중 총 이산화탄소는 중탄산염 수준을 통해 대사적 변화와 관련된 산-염기 불균형을 반영한다. 실제로 환자가 세포 수준의 대사 이상이 시작되었더라도 활력징후의 문제까지 발생하지 않게 되면 NEWS 점수에 충분히 반영되지 않을 가능성이 있다. 그러므로 본 연구 결과와 같이 환자의 생리학적 변화와 대사적 변화를 동시에 반영한 모델을 사용하면 실제 환자 급성 악화 예측률을 더욱 향상시킬 수 있을 것이다.

본 연구 결과, 비계획적 중환자실 입실 예측에서 확장모델의 민감도가 3.5%p 증가하였다. 증가폭이 작긴하지만, 실제 임상에서 신속대응팀이 급성 악화 고위험군을 선별함에 있어 NEWS 단독모델을 사용할 때보다 확장모델을 사용할 때 더 많이 조기 선별할 수 있는 근거를 제공할 수 있다는 점에서 의미가 있다. 반면 확장모델의 민감도가 59.3%라는 것은 선택된 절단점에서 비계획적 중환자실 입실 환자의 약 40.7%가 선별되지 않을 수 있음을 의미하기 때문에 본 모델을 활용함에 있어 고위험 환자를 선별하고 우선순위를 결정하기 위한 보조적 도구로 활용하는 것이 적절할 것이다. 한편, 비계획적 중환자실 입실 예측의 특이도는 5.1%p 증가하였다. 이는 중환자실에 입실하지 않는 환자 100명당 약 5명의 위양성 경보를 줄일 수 있음을 의미한다. 선행연구에 따르면 일반병동에서 NEWS 기반 경보를 적용한 결과 경보 후 12시간 이내에 실제 급성 악화와 같은 임상사건이 발생한 비율은 낮은 수준에 불과하다고 보고하고 있다[37]. 이렇게 환자 특성을 반영하지 않은 낮은 성능의 자동경보는 의료진들에게 경보 피로를 유발하고 임상적 대응체계를 약화시킬 수 있다고 지적하였다[37]. 그러므로 본 확장모델의 특이도 향상은 경보의 효율성을 높여 신속대응팀의 조기 개입을 수월하게 하는데 기여할 가능성이 있음을 보여준다.

본 연구 결과, 7일 이내 사망 예측에서 확장모델의 특이도가 19.2%p로 증가하였다. 이러한 결과는 사망 위험이 낮은 환자가 고위험군으로 분류될 가능성을 줄여 신속대응팀이 상대적으로 사망 위험이 높은 환자를 우선적으로 선정하는데 상당한 기여를 할 수 있을 것으로 판단된다.

본 연구는 단일 기관에서 수행한 후향적 연구로 연구 결과를 다른 의료기관이나 다른 환자 집단에 일반화하는데 제한이 있다. 또한 전체 대상자 934명 중 혈중 젖산과 혈중 총 이산화탄소 검사 결과가 없는 289명을 제외하였는데, 해당 검사는 환자 상태와 임상의 판단에 따라 선택적으로 시행되었을 가능성이 있어 분석 대상이 전체 신속대응팀 활성화 환자를 충분히 대표하지 못했을 수도 있다. 아울러 연명의료중단 및 심폐소생술 거절 대상자를 제외한 것은 7일 이내 사망 예측모형에서 선택 편향을 초래하였을 가능성이 있기에 본 연구 결과를 해석하고 다른 환자 집단에 적용할 때 주의가 필요하다. 또한 동일한 자료를 이용하여 예측모형을 개발하고 성능을 평가하였으며, bootstrap validation이나 교차검증과 같은 내부검증을 수행하지 못하였다. 따라서 본 연구에서 제시한 예측성능은 다소 낙관적으로 추정되었을 가능성이 있으며, 향후 독립된 자료를 이용한 내부 및 외부검증이 필요하다. 또한 본 연구는 회귀모형을 개발하고 AUROC를 통해 성능을 평가함에 있어 bootstrap validation이나 교차검증과 같은 내부검증을 수행하지 못하였다. 따라서 본 연구에서 제시한 예측성능은 다소 낙관적으로 추정되었을 가능성이 있으며, 향후 독립된 자료를 이용한 내부 및 외부 검증이 필요하다.

## 결론

본 연구는 일반병동에 입원한 환자를 대상으로 NEWS, 혈중 젖산, 혈중 총 이산화탄소가 비계획적 중환자실 입실 및 7일 이내 사망 간의 관련성을 분석하였다. 더불어 혈중 젖산과 혈중 총 이산화탄소를 NEWS에 포함한 확장 모델을 통해 비계획적 중환자실 입실과 7일 이내 사망 예측에 있어 NEWS 단독 모델보다 향상된 예측력을 보이는지를 평가하였다. 연구 결과, 세 변수 모두 독립적인 예측 요인으로 확인되었으며, NEWS 단독보다 혈중 젖산 및 혈중 총 이산화탄소를 함께 활용한 확장 모델이 더 높은 예측력을 보였다.

특히, NEWS는 활력징후를 기반으로 한 간편한 조기경고점수이며, 혈중 젖산과 혈중 총 이산화탄소는 조직 저산소증과 산-염기 불균형상태를 반영하는 지표로서 상호 보완적 역할을 하였다. 이러한 결과는 환자 상태 악화를 조기에 감지하고 중재할 수 있는 객관적인 근거를 제공하며, 임상 현장에서 세 가지 지표를 통합적으로 활용하는 것이 환자 악화를 조기에 인지하는 데 유용함을 보여준다.

따라서 본 연구는 NEWS에 혈중 젖산, 혈중 총 이산화탄소를 포함한 확장 모델의 활용이 일반병동 입원환자의 급성 악화 예측을 높이는 새로운 지표로서의 역할 가능성을 지지하며, 향후 새로운 조기경고점수를 개발하는데 기초자료로 활용될 수 있을 것이다.

본 연구 결과를 통해 다음을 제언하고자 한다.

첫째, 신속대응팀 활성화 환자의 NEWS, 혈중 젖산, 혈중 총 이산화탄소와 비계획적 중환자실 입실 및 7일 이내 사망 간의 관련성을 확인하기 위해 반복 연구를 제언한다. 본 연구는 단일 기관의 대상자를 대상으로 단기간에 수행된 연구로, 결과의 일반화에 한계가 있다. 따라서 다기관 및 장기간에 걸친 반복 연구를 통해 본 연구 결과의 타당성을 검증할 필요가 있다.

둘째, 전자의무기록시스템과 연동된 NEWS, 혈중 젖산, 혈중 총 이산화탄소 기반의 자동 스크리닝 체계를 마련하여, 고위험 환자에 대해 신속대응팀의 적극적인 조기 개입이 이루어질 수 있는 환경을 조성하는 것이 바람직하다.

셋째, 본 연구는 NEWS, 혈중 젖산, 혈중 총 이산화탄소가 모두 존재하는 환자만을 대상으로 하였기 때문에, 해당 검사 항목이 누락된 환자들은 분석에서 제외되었다. 이에 따라 연구 대상 선정에 제한이 있었으며, 이는 연구 결과의 일반화에 한계를 줄 수 있다. 향후 연구에서는 다양한 임상 데이터를 포괄하거나, 측정에서 누락된 데이터를 보완할 수 있는 방법론적 접근이 필요하다.

넷째, 본 연구는 신속대응팀을 활성화함에 있어 스크리닝에 의한 군과 의료진 호출에 의한 군을 구분하지 않고 통합하여 분석하였다. 이에 따라 각 활성화 방식의 특성과 개입 시점의 차이를 충분히 반영하지 못했을 가능성이 있으며, 이는 예측모형 성능에 영향을 미쳤을 가능성을 배제할 수 없다. 향후 연구에서는 활성화 유형을 구분하여 환자 예후를 비교하여 분석하는 접근이 필요하다.

## Figures and Tables

**Table 1.** Comparison of Unplanned ICU Admission According to General Characteristics, NEWS, Blood Lactate, Serum Total CO_2_ (N = 437)

Characteristics	Classification	Admission (n = 145)	Non-admission (n = 292)	χ^2^, t or U	*p*
		n (%) or M ± SD or Median (IQR)			
Sex	Men	87 (33.7)	171 (66.3)	0.08	.773
	Women	58 (32.4)	121 (67.6)		
Age (years)		69.77 ± 14.13	69.46 ± 13.80	0.22	.825
Department of medicine	Medical	131 (34.2)	252 (65.8)	1.46	.227
	Surgical	14 (26.0)	40 (74.0)		
Underlying disease^†^	Cardiovascular disease	74 (33.8)	146 (66.2)	0.07	.786
	Malignancy	54 (29.5)	129 (70.5)	1.91	.166
	Diabetes mellitus	48 (34.5)	91 (65.5)	0.16	.682
	Chronic lung disease	43 (33.1)	87 (66.9)	0.001	.976
	Cerebrovascular disease	31 (33.7)	61 (66.3)	0.01	.906
	Chronic kidney disease	29 (38.2)	47 (61.8)	1.02	.311
	Hepatobiliary disease	20 (43.5)	26 (56.5)	2.45	.117
	Gastrointestinal disease	16 (39.0)	25 (61.0)	0.69	.400
	Thyroid disease	4 (21.1)	15 (78.9)	1.31	.250
Evaluation of activation^‡^	Respiratory distress	56 (27.3)	149 (72.7)	40.90	< .001
	Metabolic acidosis	35 (57.4)	26 (42.6)		
	Hypotension	18 (37.5)	30 (62.5)		
	Altered mental status	6 (15.0)	34 (85.0)		
	Arrhythmia	4 (12.5)	28 (87.5)		
	Sepsis/Septic shock	18 (58.1)	13 (41.9)		
	Shock	8 (40.0)	12 (60.0)		
NEWS^§^		9.00 (6.00-11.00)	6.00 (5.00-9.00)	13,230.50	< .001
Blood lactate (mmol/L)^§^		2.17 (1.06-4.12)	1.44 (0.90-2.09)	15,665.00	< .001
Serum total CO_2_ (mmol/L)^§^		20.00 (17.00-23.50)	22.00 (19.00-25.00)	16,108.00	< .001

M = Mean; SD = Standard deviation; IQR = Interquartile range; NEWS = National Early Warning Score.

^†^Multiple responses; Unadjusted *p* values are presented, and statistical significance was determined using a Bonferroni-adjusted α of .006; ^‡^Fisher-Freeman-Halton exact test; ^§^Mann-Whitney U test.

**Table 2.** Comparison of 7-Day Mortality According to General Characteristics, NEWS, Serum Lactate, and Serum Total CO_2_ (N = 437)

Characteristics	Classification	Death (n = 41)	Survival (n = 396)	χ^2^, t or U	*p*
		n (%) or M ± SD or Median (IQR)			
Sex	Men	29 (11.2)	229 (88.8)	2.55	.110
	Women	12 (6.7)	167 (93.3)		
Age (years)		68.80 ± 12.32	69.64 ± 14.06	0.36	.714
Department of medicine^†^	Medical	41 (10.7)	342 (89.3)	6.37	.012
	Surgical	-	54 (100.0)		
Underlying disease^‡^	Cardiovascular disease	21 (9.6)	198 (90.4)	0.02	.882
	Malignancy	22 (12.0)	161 (88.0)	2.58	.108
	Diabetes mellitus	19 (13.7)	120 (86.3)	4.40	.036
	Chronic lung disease	11 (8.5)	119 (91.5)	0.18	.668
	Cerebrovascular disease	9 (9.8)	83 (90.2)	0.02	.882
	Chronic kidney disease	4 (5.3)	72 (94.7)	1.83	.175
	Hepatobiliary disease	7 (15.2)	39 (84.8)	2.05	.151
	Gastrointestinal disease	1 (2.4)	40 (97.6)	2.56	.109
	Thyroid disease	-	19 (100.0)	2.05	.152
Evaluation of activation^§^	Respiratory distress	16 (7.8)	189 (92.2)	9.29	.157
	Metabolic acidosis	12 (19.7)	49 (80.3)		
	Hypotension	3 (6.2)	45 (93.8)		
	Altered mental status	3 (7.5)	37 (92.5)		
	Arrhythmia	2 (6.3)	30 (93.7)		
	Sepsis/Septic shock	3 (9.7)	28 (90.3)		
	Shock	2 (10.0)	18 (90.0)		
NEWS^∥^		9.00 (7.00-11.00)	7.00 (5.00-9.00)	5,212.00	< .001
Blood lactate (mmol/L)^∥^		2.60 (1.48-5.38)	1.46 (0.90-2.36)	4,690.50	< .001
Serum total CO_2_ (mmol/L)^∥^		18.00 (15.00-22.00)	22.00 (19.00-25.00)	5,129.50	< .001

M = Mean; SD = Standard deviation; IQR = Interquartile range; NEWS = National Early Warning Score.

† Fisher’s exact test; ^‡^Multiple responses; Unadjusted *p* values are presented, and statistical significance was determined using a Bonferroni-adjusted α of .006; ^§^Fisher-Freeman-Halton exact test; ^∥^Mann-Whitney U test.

**Table 3.** Factors Associated with Unplanned ICU Admission and 7-Day Mortality

	Unplanned ICU Admission										7-Day Mortality									
	Model 1					Model 2					Model 1					Model 2				
	B	SE	Wald	OR (95% CI)	*p*	B	SE	Wald	OR (95% CI)	*p*	B	SE	Wald	OR (95% CI)	*p*	B	SE	Wald	OR (95% CI)	*p*
NEWS	0.22	0.04	38.69	1.25 (1.16-1.34)	< .001	0.21	0.04	31.63	1.23 (1.14-1.32)	< .001	0.19	0.05	14.46	1.20 (1.09-1.32)	< .001	0.16	0.05	9.55	1.17 (1.06-1.29)	.002
Blood lactate (mmol/L)						0.19	0.06	9.77	1.22 (1.08-1.37)	.002						0.15	0.06	5.66	1.16 (1.03-1.31)	.017
Serum total CO_2 (mmol/L)_						-0.06	0.02	6.79	0.94 (0.90-0.99)	.009						-0.09	0.04	6.75	0.91 (0.85-0.98)	.009
-2 log likelihood	510.50					483.40					257.47					236.18				
Cox & Snell R²	.10					.15					.03					.08				
Nagelkerke R²	.14					.21					.07					.17				
Hosmer–Lemeshow	.89					.61					.52					.13				
EPV	145.00					48.33					41.00					13.67				

Model 1 = only NEWS model; Model 2 = extended NEWS model (NEWS + serum lactate + serum total CO_2_). ICU = Intensive care unit; SE = Standard error; OR = Odds ratio; CI = Confidence interval ; NEWS = National Early Warning Score; EPV = Events per variable.

**Table 4.** Predictive Performance of the Extended NEWS Model for Unplanned ICU Admission and 7-Day Mortality

	Unplanned ICU admission					7-Day Mortality				
	Cut off	Sensitivity	Specificity (%)	AUROC (95% CI)	*p* for difference	Cut off	Sensitivity (%)	Specificity (%)	AUROC (95% CI)	*p* for difference
Model 1	8.50	55.8	74.0	.69 (.63-.74)	.009	6.50	80.5	44.9	.68 (.60-.76)	.003
Model 2	0.37^†^	59.3	79.1	.74 (.69-.79)		0.08^†^	82.9	64.1	.77 (.71-.83)	

Predicted probabilities were calculated using the following logistic regression equations: Unplanned ICU admission Model 1 logit(p)=-2.40+0.22(NEWS), Model 2 logit(p)=-1.51+0.21(NEWS)+0.19(Lactate)-0.06(Total CO_2_), 7 Day Mortality Model 1 logit(p)=-3.78+0.19(NEWS), and Model 2 logit(p)=-2.10+0.16(NEWS)+0.15(Lactate)-0.09(Total CO_2_). ICU = Intensive care unit; AUROC = Area under the receiver operating characteristic curve; CI = Confidence interval; NEWS = National Early Warning Score.

† Predicted probability cutoff.

## Appendix 1.

Flow chart of case enrollment.

## Appendix 2.

National Early Warning Score (NEWS)
