# Supplementary material for: Predictive performance of an extended NEWS model for acute deterioration in general ward inpatients: a retrospective observational study
Source: J Korean Biol Nurs Sci. 2026 Aug 26;28(3):515–27. [Article in Korean] doi: 10.7586/jkbns.26.031 (PMC13533601; doi:10.7586/jkbns.26.031)
Supplement: Supplementary file 1 [file jkbns-26-031-Appendix-1.pdf]

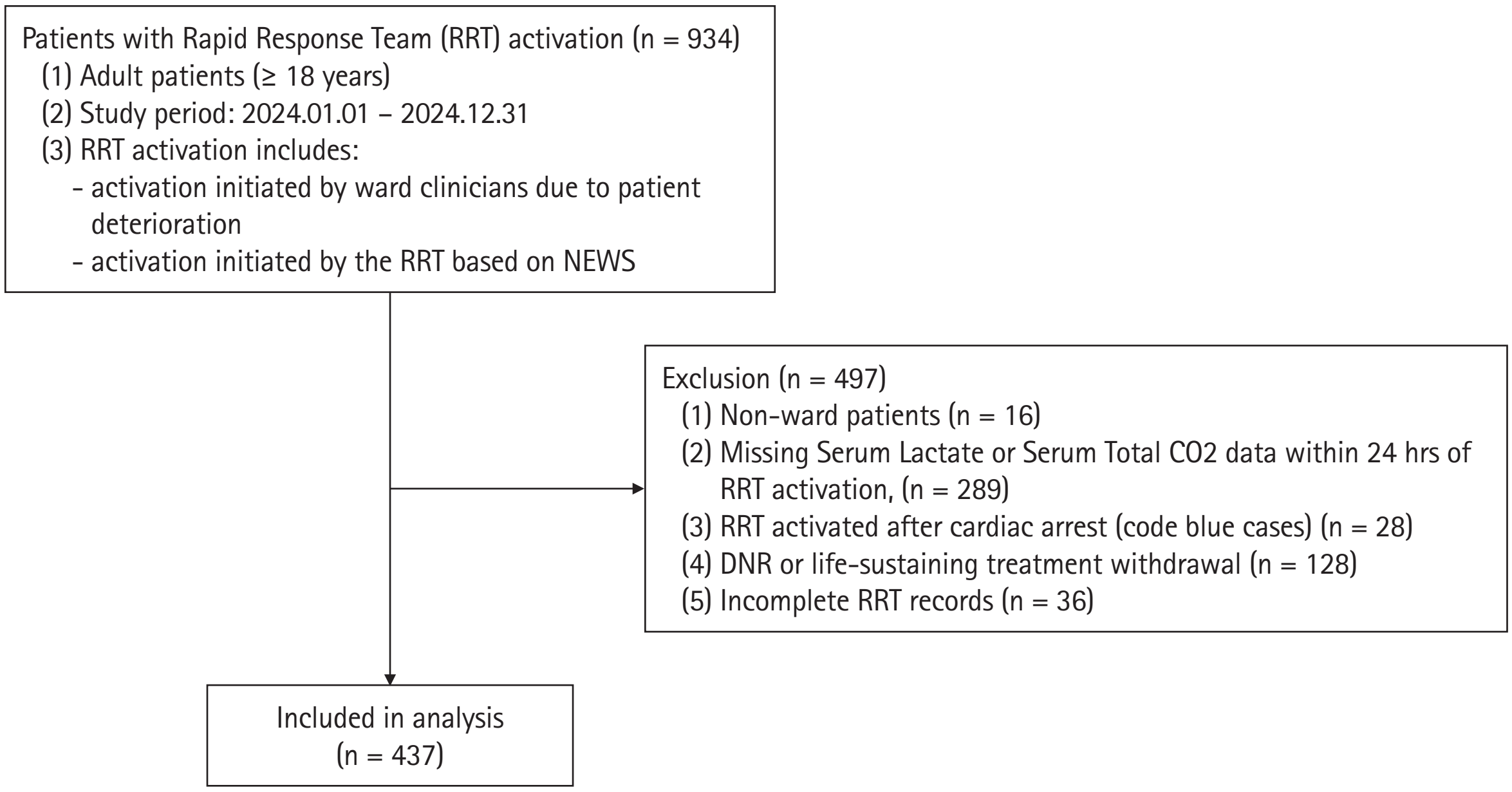

NEWS = National Early Warning Score.

Appendix 1. Flow chart of case enrollment.

Appendix 2. National Early Warning Score (NEWS)

Parameters	3	2	1	0	1	2	3
Respiration rate (/min)	≤ 8		9-11	12-20		21-24	≥ 25
Oxygen saturations (%)	≤ 91	92-93	94-95	≥ 96			
Any supplemental oxygen		Yes		No			
Temperature (°C)	≤ 35.0		35.1-36.0	36.1-38.0	38.1-39.0	≥ 39.1	
Systolic blood pressure (mmHg)	≤ 90	91-100	101-110	111-219			≥ 220
Heart rate (/min)	≤ 40		41-50	51-90	91-100	111-130	≥ 131
Level of consciousness				A			V,P,U

A= Alert; V = Verbal; P = Pain; U = Unresponsive.
